# Audio recordings dataset of genuine and replayed speech at both ends of a telecommunication channel

**DOI:** 10.1016/j.dib.2020.106652

**Published:** 2020-12-17

**Authors:** Wei Shang, Maryhelen Stevenson

**Affiliations:** Department of Electrical and Computer Engineering, University of New Brunswick, Canada

**Keywords:** UNB database, Replay/playback recordings, Audio fingerprinting, Automatic speaker verification, Playback attacks, Replay attacks, Spoofing attacks

## Abstract

The recordings in this database were collected for the purpose of evaluating the ability of a copy-detection based playback attack detector to safeguard a remote-access speaker-verified and passphrase-protected system from playback attacks. The database includes multiple utterances of the same phrase by the same person in addition to a variety of distorted versions of many of the utterances.  Multiple distortions of an utterance were obtained, in part, by simultaneously recording the utterance at both ends of a telecommunication channel – using a digital voice recorder to obtain the user-end (*i.e.*, in-person) recording and a telephony board to obtain the system-end recording.  While the former suffers little distortion, the latter suffers the “non-stationary” distortion imposed by the channel.  Additional distortions of the same utterance were captured at the system-end of the channel when the in-person recording was replayed at the user-end; these additional recordings simulate playback attacks and suffer the distortion imposed by both the playback device and the channel.  The database may be used: to evaluate the vulnerability of a speaker verification system (SVS) to playback attacks; to evaluate the performance of a copy-detection or distortion-detection based playback attack detector (PAD); to evaluate the overall security of a speaker verification system in tandem with a playback attack countermeasure; or to investigate the distortion imposed by various telecommunication channels and/or playback speakers.

## Specifications Table

**Table:** 

Subject	Signal Processing
Specific subject area	Audio fingerprinting of speech signals, copy-detection based playback attack detection
Type of data	Digital audio files
How data were acquired	Intruder recordings are recorded using an OLYMPUS WS-100 Digital Voice Recorder at the user/transmitting-end of a telecommunication channel. Authentic and playback recordings are collected using a Dialogic D/21H telephony board at the system-end (*i.e.*, the receiving end) of the telecommunication channel.
Data format	Raw audio files in WAV format
Parameters for data collection	Participants, passphrases, playback devices and telecommunication channel types are the controlled parameters during the collection process. All recordings were made in environments with minimal background noise.
Description of data collection	The participant called into a system via a telecommunication channel of their choosing, where their utterance was recorded as an authentic recording. In certain cases, their utterance was also recorded, using a digital voice recorder (DVR), at the participant's location; the resulting recording is called an intruder recording. Subsequently, the researcher played back the intruder recording via a telecommunication channel to the same system, where it was recorded as a playback recording.
Data source location	Institution: University of New Brunswick City/Town/Region: Fredericton Country: Canada
Data accessibility	Repository name: Audio recordings of genuine and replayed speech at both ends of a telecommunication channel Data identification number: http://dx.doi.org/10.17632/5t56sjbgf6.1 Direct URL to data: http://dx.doi.org/10.17632/5t56sjbgf6.2
Related research article	Wei Shang, Maryhelen Stevenson, Detection of speech playback attacks using robust harmonic trajectories, Computer Speech & Language, 65, https://doi.org/10.1016/j.csl.2020.101133

**Value of the Data**

• The database allows for the design and evaluation of a feature set that is not only capable of distinguishing between distinct utterances of the same phrase by the same speaker but is also capable of recognizing when two recordings contain differently distorted versions of the same utterance. Such feature sets are essential for copy-detection based playback attack detectors.
• The database may be used by researchers attempting: to evaluate vulnerabilities of speaker verification systems to playback attacks; to evaluate copy-detection or distortion-detection based approaches to playback attack detection in speaker verification; or to assess the performance of an audio fingerprinting technique in application to short-duration speech signals.
• With many factors considered during the collection of the recordings (*e.g.*, type of communication channel, type and quality of playback device, differences in phrase, difference in speaker, *etc.*), the database may be used to gain preliminary insight regarding the performance impact of these factors.

## 1 Data Description

**Fig. 1:**
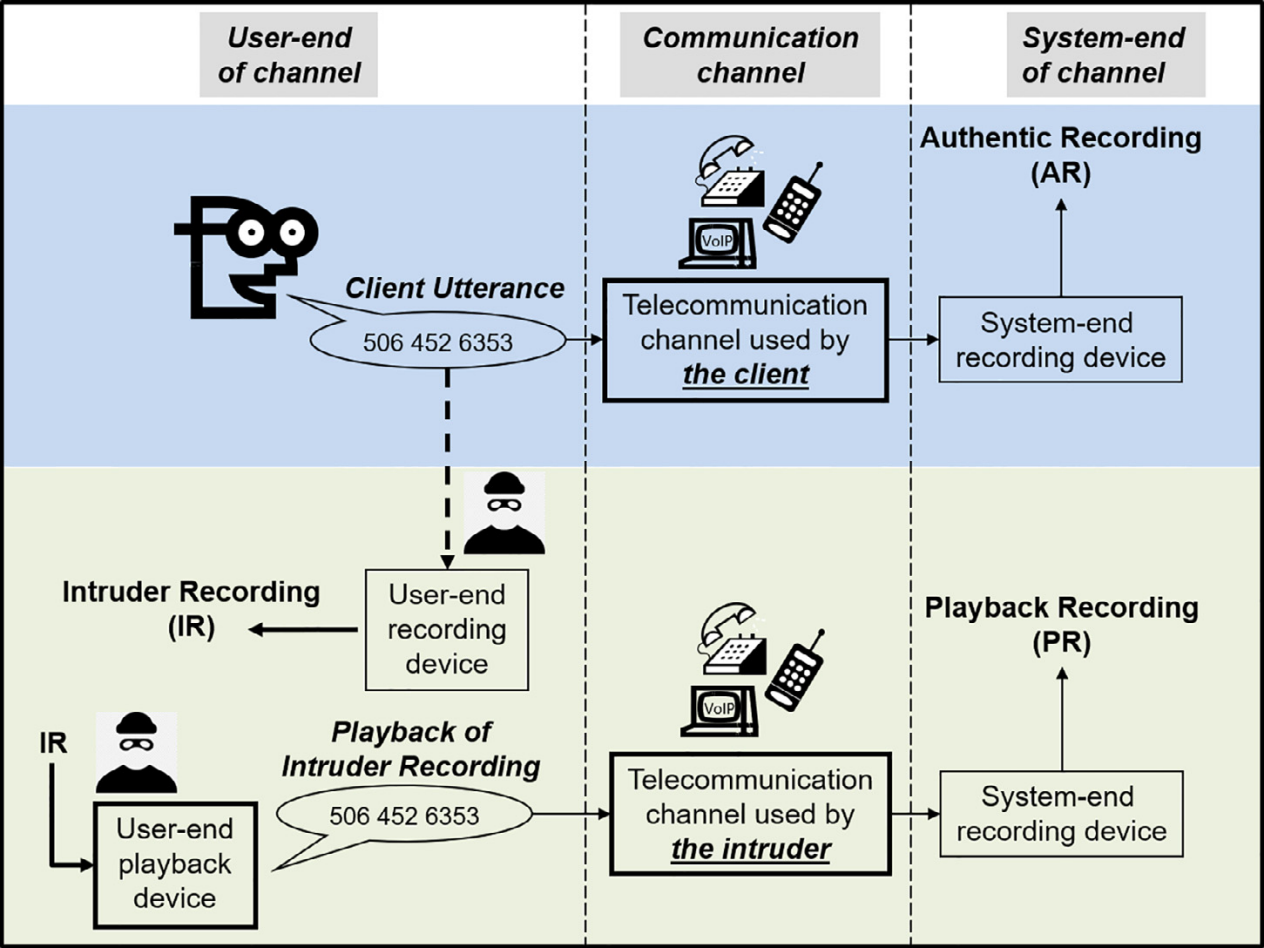
Illustration of the simulated scenarios and the associated recording types.

The database consists of three types of recordings: intruder recordings, authentic recordings and playback recordings.  The names of these recordings are based on the two scenarios simulated by the data collection process and depicted in Fig. 1; both scenarios involve attempts to gain entry to a remote-access speaker-verified and passphrase protected system.  The upper (blue) panel depicts the normal-use scenario where a client connects to the system via a telecommunication channel of their choosing and utters their passphrase.  The lower (green) panel depicts a two-step playback-attack scenario: in the first step, the intruder clandestinely obtains a recording (referred to as an intruder recording) of the true client uttering their passphrase while attempting to gain access to the system; in the second step, the intruder connects to the system via a telecommunication channel of his/her choosing, then uses a playback device to replay the intruder recording. In both scenarios, and as part of the system's security protocol, the speech signal that originates from the user-end of the channel is recorded, following transmission, at the system-end of the channel.  System-end recordings that result from the normal-use scenario are referred to as authentic recordings; whereas, those that result from the attack scenario are referred to as playback recordings.  Finally, with reference to Fig. 1, we note that: the line from the client's utterance down to the intruder's recording device is dashed so as to emphasize that intruder recordings are not always made; and the boxes enclosing the user-end playback device and the telecommunication channel are drawn with thick borders so as to indicate that there are several options for playback-device and channel type.

The recording database contains 4680 recordings [1], with details of each recording included in the metadata file (also available in [1]).  Each recording in the database is uniquely identified by a five-digit recording ID, which is also used as the file name of the recording. Furthermore, each recording captures one of 1080 utterances (note: each of 4 participants uttered each of 3 phrases a total of 90 times) – with each utterance being uniquely identified by a five-digit number. In some cases (corresponding to recording sessions that mimic the normal-use scenario when no intruder recording is made), the utterance will be solely captured in one authentic recording.  In other cases, multiple recordings capture the same utterance and, thus, share the same utterance ID; for example, the utterance captured (at the user-end) in an intruder recording is the same as that captured (at the system-end) in the corresponding authentic recording; furthermore, the same utterance is also captured in 9 playback recordings (one for each of the 9 combinations of playback device and telecommunication channel) that are generated from the intruder recording.  Although multiple recordings may capture the same utterance, the distortion affecting the utterance will differ in each recording; the types of distortion affecting the utterance captured in a specific recording may be deduced from the following group of recording attributes: RECORDING TYPE; CHANNEL TYPE (in the case of an authentic or playback recording); and PLAYBACK DEVICE (in the case of a playback recording). The complete list of recording attributes is found in Table 1.

**Table 1:** Descriptions of attributes used in the metadata file.

Column name	Description
Recording ID	A five-digit number uniquely identifying the recording (also used as the filename for the recording), unique across the entire database
Recording type	One of three types of recordings: Intruder, Authentic, and Playback. Differences between recording types include the source of the audio signal (human or recording) at the user-end of the channel, and the location where the recording is made (user-end or system-end of the channel). An illustration of their collection is depicted in Fig. 2. **Intruder recording**: recording of the client's utterance as captured at the client's location on the user-end of the channel **Authentic recording**: recording of the client's utterance that was spoken on the user-end of the channel and captured, following transmission, at the system-end of the channel **Playback recording**: recording of the audio signal generated from a playback device on the user-end of the channel and captured, following transmission, at the system-end of the channel.
Utterance ID	a five-digit number which uniquely identifies the utterance captured in the recording, multiple recordings may capture the same utterance, but the distortion affecting the utterance will differ in each recording
Client ID	a single character (A, B, C, or D) identifying the client/participant. Characteristics of the clients: **A**: male, native English speaker **B**: male, non-native English speaker **C**: female, native English speaker **D**: female, non-native English speaker
Phrase ID	a single character (T, U, or P) identifying the phrase that was uttered: **P** (postal code): “E3B 5A3” **T** (telephone number): “506 452 6353” **U** (university name): “University of New Brunswick”
With-IR (applicable only to authentic recordings)	A Boolean value indicating the existence of an intruder recording (IR) that captures the same utterance **True**: the utterance captured in the authentic recording is also captured in an intruder recording **False**: the utterance captured in the authentic recording is not captured in any intruder recording
Channel type (applicable only to authentic and playback recordings)	a single character (C, L, or V) indicating the type of telecommunication channel through which the audio signal was transmitted from the user-end to the system-end, where the recording (either authentic or playback) was made. **C**: cellular **L**: landline **V**: Voice-over-IP
PLAYBACK DEVICE (applicable only to playback recordings)	a single character (D, C, S) indicating the type of speaker through which the intruder recording is replayed, at the user-end, when making playback recordings. (The resulting audio signal is picked up by the input microphone to a telecommunication channel and transmitted to the system-end where it is recaptured as a playback recording). **D**: built-in DVR speaker (very low quality, shown on left side of Fig. 3) **C**: external computer speaker (medium quality, shown in middle of Fig. 3) **S**: stereo speaker (fairly good quality, shown on right side of Fig. 3)

Table 2 shows sample entries from the metadata file, which contains the values for all the recording attributes defined in Table 1. Please note that cells are left as empty if the column is not applicable to the recordings. Specifically: CHANNEL TYPE is only applicable to authentic and playback recordings; WITH-IR is only applicable to authentic recordings, and PLAYBACK DEVICE is only applicable to playback recordings.

**Table 2:** Sample entries in the metadata file.

Rec. ID	Rec. type	Utter. ID	Client ID	Phrase ID	Channel type	With-IR	Playback device
⋮							
00391	authentic	00091	A	T	C	False	
00392	authentic	00092	A	T	C	False	
00393	authentic	00093	A	T	C	False	
⋮							
00449	authentic	00149	A	T	V	False	
00450	authentic	00150	A	T	V	False	
00451	intruder	00151	A	T			
00452	authentic	00151	A	T	C	True	
00453	playback	00151	A	T	C		D
00454	playback	00151	A	T	L		D
00455	playback	00151	A	T	V		D
00456	playback	00151	A	T	C		C
00457	playback	00151	A	T	L		C
00458	playback	00151	A	T	V		C
00459	playback	00151	A	T	C		S
00460	playback	00151	A	T	L		S
00461	playback	00151	A	T	V		S
00462	intruder	00152	A	T			
00463	authentic	00152	A	T	C	True	
00464	playback	00152	A	T	C		B
⋮							

The complete recording data set of 4680 recordings can be equally divided among twelve sets of 390 recordings, with each set pertaining to a particular client and passphrase (CaP) combination (as determined by the CLIENT ID and PHRASE ID). Each of the 390 recordings in a CaP set captures a uniquely distorted version of one of 90 unique utterances of the specified passphrase by the specified client. Of the 90 unique utterances:

• 60 of the utterances are captured exclusively by one of 60 authentic recordings (20 for each of the three channel types, C, L and V). For example, recordings 00391–00450 in Table 2 correspond to the 60 authentic recordings containing distinct utterances of passphrase T by client A – that were not captured in an intruder recording (as indicated by a value of False for the attribute WITH-IR).
• 30 of the utterances are captured by multiple recordings. In particular, each of these utterances is captured in eleven recordings (*e.g.*, utterance 00151 is captured in recordings 00453–000461) which include: one intruder recording, one authentic recording, and nine playback recordings (one for each of the nine CHANNEL TYPE and PLAYBACK DEVICE combinations). Note that while both playback recordings 00458 and 00460 are captured at the system-end when intruder recording 00451 is played back at the user-end, the former is played back using an external computer speaker through a VoIP channel while the latter is played back using a stereo speaker through a landline. The same client utterance is captured in the corresponding authentic recording (*i.e.*, recording 00452), which captures a version of the utterance that has been distorted by its transmission through a cellular channel but has not been distorted by a playback device. In all, these 30 utterances are captured in 330 recordings: 30 intruder recordings, 30 authentic recordings (10 for each of the three channel types), and 270 playback recordings.

## 2 Experimental Design, Materials and Methods

The design of the collection process is largely driven by the development of a copy-detection based playback attack detector, whose goal is to detect playback attacks by detecting that the system-end recording of the playback attack access attempt captures the same utterance as captured by a system-end recording of a previous access attempt (stored in the system).  As such, the design decisions depend on the list of factors that are perceived to be most impactful to the performance of the PAD, namely: participants, passphrases, telecommunication channels and playback devices [2].

The collection process consisted of two tracks, with each targeting specific types of recordings. In the client-track, the participants (here on referred to as clients) were given specific steps to follow when providing speech samples for the authentic recordings, and if applicable, the intruder recordings as well; in the intruder-track, the researcher carried out the task of making playback recordings by systematically playing back the available intruder recordings (resulting from the client-track). Details of the setups and steps for each track are provided below.

In addition, an Interactive Voice Response (IVR) application was implemented to work with the telephony board and automate the labelling of the recordings received over the telecommunication channel. Once connected, the application prompts the caller to enter a sequence of digits that encodes the metadata specific to the recording (*e.g.*, client id, channel type, *etc.*); after the call ends, the application saves the recordings with appropriate file names, thus allowing easy association of the different recordings originating from the same utterance.

### 2.1 The client-track

Over the span of four months in 2007, four clients partook in 90 recording sessions. Client A and C are male, whereas client B and D are female; client A and B are native English speakers, whereas client C and D are non-native English speakers. For each recording session, the client connected to the system, from a location of their choosing, using one of three telecommunication-channel types (landline, cellular, or VoIP) and uttered three different passphrases (a telephone number: “506 452 6353”; a phrase: “University of New Brunswick”; and a postal code: “E3B 5A3”) while keeping the channel-input microphone approximately one inch from the side of the mouth. The passphrases (all associated with the office on campus where the system-end recordings were made) were kept the same for the four clients so as to allow meaningful comparison of the results across different clients for the same passphrase, or across different passphrases for the same client. The utterances were recorded at the other end (*i.e.*, the system-end of the channel) using a computer equipped with a Dialogic D/21H telephony board (8 bits per sample at a sampling rate of 8 kHz). The recordings were manually cropped to include only one passphrase per recording. This resulted in a total of 90 authentic recordings for each of the 12 client and passphrase (CaP) combinations (30 for each of the three channel types).

**Fig. 2:**
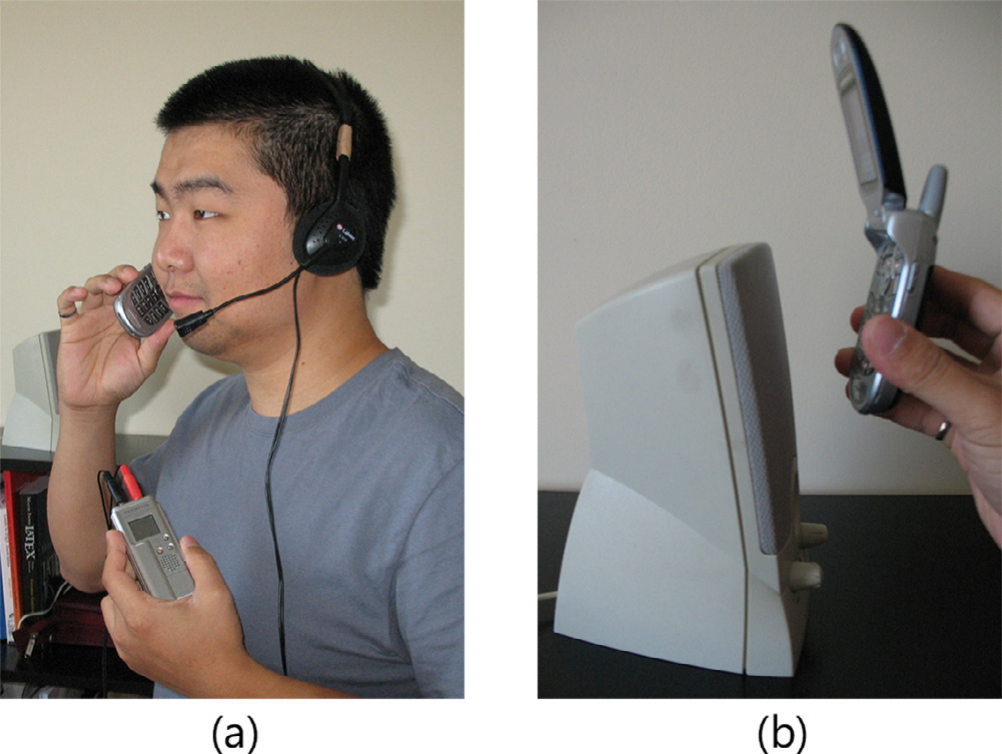
Illustration of the user-end activity when generating various recordings, sources are approximately one inch from the microphones. (a): simultaneous generation of intruder (via headset connected to DVR) and authentic recordings (via channel input microphone); (b): generation of playback recordings.

While ideally collecting both authentic and intruder recordings for each utterance is preferred, doing so requires more effort from the clients; so as to lighten the burden on the clients, and therefore maximize the probability that clients complete all sessions, the intruder recordings were collected only for a subset of the recording sessions. More specifically, for 30 of the 90 recording sessions (10 sessions for each channel type), the client's utterance was also recorded at the user end of the channel using an OLYMPUS WS-100 digital voice recorder (DVR), set at a sampling rate of 44.1 kHz, so as to obtain a set of 30 intruder recordings for each CaP combination. (The 30 corresponding authentic recordings will have a value of true for WITH-IR.) During these 30 sessions, the participant kept two microphones (both the channel-input microphone as well as the microphone of a headset connected to the DVR) approximately one inch from and on opposite sides of the mouth (see Fig. 2a). Please note that the particular setup is designed to mimic the worst case scenario for the speaker verification system; in that, high quality intruder recordings are obtained, which will have a high success rate when used in playback attacks. Such a scenario is possible in the case where the access site is unsecured (*e.g.*, a public office area), which allows the intruder to easily plant a microphone in close proximity to the channel input microphone (*e.g.*, in the handset of a telephone).

### 2.2 The intruder-track

**Fig. 3:**
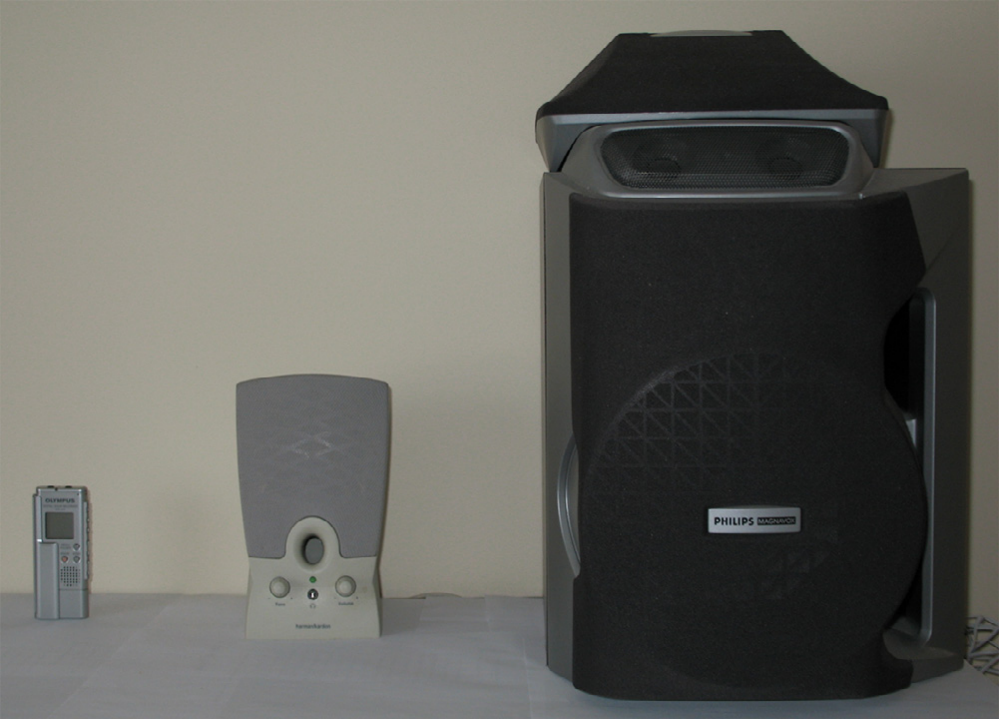
Three playback speakers of varying quality. From left to right, OLYMPUS WS-100 Digital Voice Recorder, Harman Kardon HK19.5 computer speaker, and PHILIPS FW560C stereo system.

To simulate playback attack to the speaker verification system, playback recordings were made at the system-end in the same way as the authentic recordings - the only difference occuring at the user-end – where, in lieu of the client uttering a passphrase, an intruder recording was played back through a playback device (*e.g.*, a speaker connected to the DVR) while the channel-input microphone was held approximately one inch in front of the device (see Fig. 2b). Three types of playback devices (see Fig. 3) were used in making the playback recordings: a stereo speaker (fairly good quality), an external computer speaker (medium quality), and the built-in DVR speaker (very low quality). For each of the 30 intruder recordings, nine playback recordings were made (one for each of the nine possible telecommunication-channel/playback-device combinations) resulting in a total of 270 playback recordings for each CaP combination. Note that, throughout the intruder-track, a Motorola V551 was used as the cellular phone and Skype was used for VoIP.

## 3 Ethics Statement

Informed consent to release the recordings was obtained from each participant, as well as the subject shown in Fig. 2.

## Declaration of Competing Interest

The authors declare that they have no known competing financial interests or personal relationships which have, or could be perceived to have, influenced the work reported in this article.
